# Delay in initiation of rabies post-exposure vaccination and influencing factors in Gondar zones of Ethiopia

**DOI:** 10.1371/journal.pntd.0014729

**Published:** 2026-10-13

**Authors:** Bezawit Kassaw Hailu, Yitayew Demessie, Asnakew Mulaw Berihun, Abebe Belete Bitew, Sefinew Alemu Mekonnen

**Affiliations:** 1 Department of Veterinary Biomedical Sciences, College of Veterinary Medicine and Animal Sciences, University of Gondar, Gondar, Ethiopia; 2 Department of Veterinary Pathobiology, College of Veterinary Medicine and Animal Sciences, University of Gondar, Gondar, Ethiopia; 3 Department of Veterinary Epidemiology and Public Health, College of Veterinary Medicine and Animal Sciences, University of Gondar, Gondar, Ethiopia; 4 School of Health, Faculty of Medicine and Health, University of New England, Armidale, NSW, Australia; 5 Department of Veterinary Epidemiology and Public Health, School of Veterinary Medicine, Bahir Dar University, Bahir Dar, Ethiopia; NIAID DIR: National Institute of Allergy and Infectious Diseases Division of Intramural Research, UNITED STATES OF AMERICA

## Abstract

Rabies is a viral zoonotic disease of warm-blooded animals, which is preventable through proper administration of post-exposure prophylaxis (PEP). In controlling rabies, delays in initiating post exposure vaccination (PEV) are a challenge in endemic regions. A study was conducted to assess the delay in initiation of PEV and identify associated factors in Ethiopia. Data was collected by face-to-face interviews from 260 dog bite victims or their guardians between January and June, 2025 using a questionnaire. Post exposure vaccination was considered delayed if it was initiated after 48 hours of the exposure incident. The prevalence of delayed initiation of PEV was calculated as a percentage from the number of individuals who initiated PEV after 48 hours and the total number of individuals exposed to rabies. Factors associated with delay were identified using logistic regression models. The prevalence of delayed in initiation of rabies PEV was 86.1% (224/260); 13.9% (36/260) of participants commenced vaccination within 48 hours of exposure. Severity of bite and ownership of biting animal were factors associated with the outcome. Deep bite (OR = 0.22, 95% CI: 0.07 - 0.66, P = 0.008) and bitten by stray dogs (OR = 0.51, 95% CI = 0.27 - 0.95, P = 0.033) reduced delay in initiation of PEV. Majority of respondents (88.5%) are aware of the existence, the signs (71.5%), the way of transmission (75.4%), and the fatal nature (96.5%) of rabies, and the need for PEV (95.4%). Another majority believe that the cost of the vaccine is fair and PEV is effective (95%), and and costs of the vaccination (76.2%) was not a reason for delay. In conclusion, the study outlined that respondents know many aspects of rabies control and had fewer barriers to uptake the PEV; however, the delay in initiating PEV was high. This underscores that respondents may not be aware of the risk of delay. Therefore, interventions focusing on the risk of delayed PEV initiation are needed.

## Author summary

Rabies is a viral zoonotic disease that is transmitted to humans mainly through a dog bite. The disease is entirely preventable by vaccinating vectors transmitting the rabies virus and administration of PEV. However, the PEV should be initiated as soon as possible following exposure. In Ethiopia, many people know the existence of rabies, its fatal nature, and the importance of PEV; but, death due to rabies is also high. Therefore, we conducted a questionnaire survey in individuals from the Central, North, and West Gondar Zones of the Amhara Region, Ethiopia, who were exposed to suspected rabid animals (or their guardians when victims were under 18 years of age) and sought rabies PEV at the Gondar Polyclinic,. Delay in initiating PEV was high, but respondents believe that the costs of the vaccination was fair. Delay was higher in victims bitten by owned dogs than those bitten by stray dogs, but victims initiate PEV without delay when they were bitten deep compared to shallow. Respondents knew many aspects of rabies control and had fewer barriers to uptake the PEV; but did not aware the risk of delay. Awareness creation on the risk of delay in initiating the PEV was suggested.

## Introduction

Rabies is one of the most important zoonotic diseases in Ethiopia circulating between canines, humans, and other animals. Literary rabies, also known as canine madness, primarily transmitted through dog bites [1]. Rabies is caused by a neurotropic, negative-sense, non-segmented, single-stranded RNA virus that belongs to the *Lyssavirus* genus of the *Rhabdoviridae* family and *Mononegavirales* order [2]. The disease is almost always fatal once clinical symptoms appear. In 2019, the disease caused an estimated 14,075 cases and 13,743 deaths worldwide with over 95% of human fatalities occurring in Africa and Asia particularly in sub-Saharan Africa where dog bites are the major source of transmission [3,4].

Rabies primarily affects poor and vulnerable communities, where deaths often go unreported [5]. Although rabies is fatal once clinical signs appear, the disease is entirely preventable. Prevention therefore remains the primary focus, targeting reservoir animals that transmit the virus, alongside post exposure prophylaxis for individuals potentially exposed to the rabies virus [6]. Pre-exposure vaccination is advised for high-risk groups such as veterinarians, laboratory workers, and children in rabies-endemic regions [7]. Post-exposure prophylaxis (PEP) for preventing rabies applied after a person has been exposed to the virus, typically through an animal bite or scratch [8]. The PEP measures include immediate wound washing with antiseptic chemicals, administration of rabies immunoglobulin when indicated, and a complete course of rabies post exposure vaccination (PEV) [9]. For effective prevention of rabies, PEV should be initiated as soon as possible [10]. However, factors such as limited awareness about importance of antiseptics, financial limitation and inadequate distribution of anti-rabies vaccine hinder effectiveness [11,12]. In addition, lack of epidemiologic data, operational challenges in accessing dogs for vaccination, and insufficient knowledge of dog population sizes hinder control efforts [13,14]. Other barriers include inadequate government commitment, financial constraints, and insufficient public health infrastructure, such as vaccination programs and access to PEV [15,16]. Despite efforts to control rabies, these gaps perpetuate significant health, economic, and social burdens [17,18].

Despite lack of rabies diagnosis centre, frequent epidemiological data and reporting on rabies outbreaks in Ethiopia, the disease remains a major public health concern, with dogs as the primary source of transmission [19]. For instance, dogs were responsible for 96.5% of bite cases, and 73.6% of the dogs involved were suspected to be rabid [20]. Incidence of rabies is high in the country; between September 2013 and August 2014, a total of 655 animal bite cases, between 2015 and 2019, 87 human rabies cases, all resulting in death [21] were recorded. Vulnerable groups include children, rural and isolated communities, and people in close contact with stray dog populations, and individuals working closely with animals, such as farmers and animal handlers [20,22]. Despite the availability of PEV in health centres, accessibility is limited by logistical barriers and high costs, especially in rural areas [20,23]. Rabies control efforts in Ethiopia are further hampered by inadequate laboratory capacity, insufficient surveillance, a lack of public awareness, and inadequate funding [24,25]. Additionally, previous vaccination programs face challenges related to low community involvement [26].

In Gondar zones of Ethiopia, rabies poses a significant public health challenge and consistent to what we know about rabies primarily transmitted through dog bites [27]. However, the rate of PEV remains low despite its proven effectiveness. It is believed that multiple factors contribute to this issue, including inadequate public awareness of rabies, financial barriers, and difficulties in accessing health services due to long distances [28]. Moreover, cultural misconceptions about rabies and perceived severity of dog animal may delay timely treatment-seeking and contribute to substantial public health and economic burdens [29]. We hypothesized that structural and individual-level factors, including health service accessibility, socio-demographic characteristics, and rabies-related knowledge, are associated with delayed initiation of PEV. Despite these challenges, data that describes the prevalence of delay in initiation of PEV and on factors influencing prompt PEV uptake in Ethiopia in general and in Gondar zones in particular are limited. Understanding the socio-economic, logistical, and behavioural barriers is essential for enhancing the effectiveness of rabies prevention strategies and reducing mortality rates associated with the disease. The objectives were to estimate the prevalence of delayed initiation of PEV and to identify associated factors.

## Methods

### Ethical statement

The study received ethical approval from College of veterinary medicine and animal sciences Ref. No: CVMAS 13/456/2025, Date 01/01/2025. Informed verbal consent was obtained from bitten victims or their guardians, in case of children, after giving explanation about the aim of the study and anonymity of the data analysis before interviewing volunteered participants. All data were analyzed and reported anonymously.

### Description of the study area

The study was conducted at the PolyClinic in Gondar City. The clinic provides a variety of health services to the community and is the only center offering PEV for individuals exposed to rabies in and around Gondar. Owing to its accessibility and role, the clinic serves residents of Gondar City as well as victims from surrounding zones who seek care following potential rabies exposure. The clinic give service for residents who are coming from Central, North, and West Gondar zones of the Amhara regional state, located in northwestern Ethiopia (Fig 1). In terms of administrative divisions, Central Gondar Zone encompasses thirteen districts, North Gondar Zone consists of seven, and West Gondar Zone consists of six districts [30]. As a centralized service point, it requires individuals to travel from both urban and rural areas to Gondar Polyclinic to access PEV.

**Fig 1:**
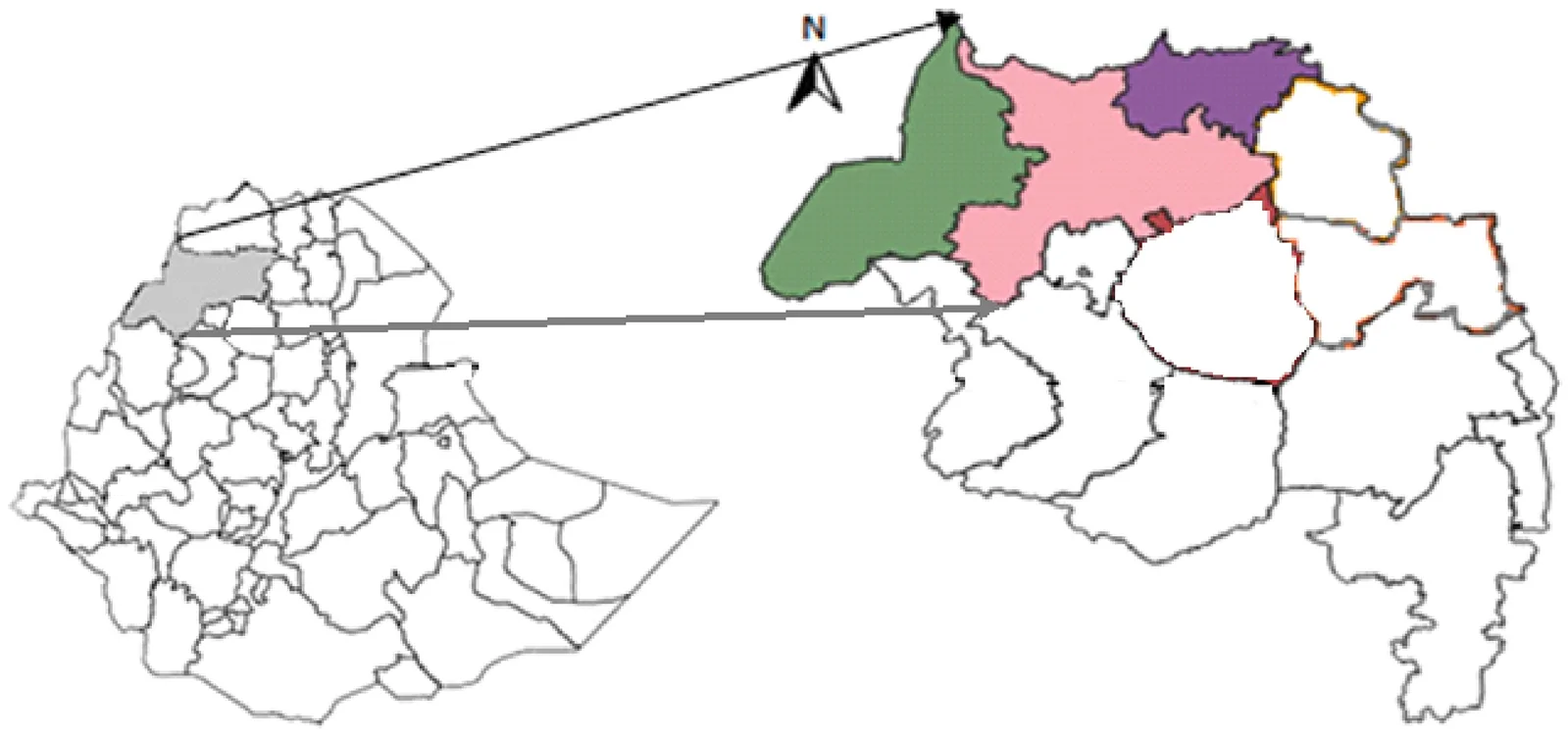
Map showing the study area (Adapted from Mengie et al. [31].

### Study population and participants

This study included individuals from the Central Gondar, North Gondar, and West Gondar Zones of the Amhara Region, Ethiopia, who had been exposed to suspected rabid animals. Study participants consisted of 260 individuals of all age groups with a history of suspected rabies exposure who sought rabies PEV at Gondar Polyclinic during the study period. Gondar Polyclinic is one of the public health centers providing rabies PEV in the area and serves exposed individuals referred from Gondar City and surrounding districts. The age distribution of study participants ranged from children to older adults.

### Study design and sample size

A cross-sectional study was conducted to estimate delay in initiating rabies PEV and to identify potential influencing factors. It was questionnaire-based conducted from January to June 2025. Delay in seeking PEV was considered if initiation of the vaccination was made after 48 hours of exposure incident, and was considered as a dependent variable. Other variables supposed to influence the initiation of PEV were tested for association with the dependent variable. As there was no research that assessed delayed initiation of rabies PEV in Ethiopia, the sample size could not be determined based on formal sample size calculation based on earlier studies. Thus, the sample size was determined by the total number of eligible cases available during the study period taking time and logistical considerations into account rather than by a priori statistical sample size calculation. However, we have considered to include 15–20 observations for each variable evaluated in the study.

### Questionnaire preparation

The study employed a structured, interviewer-administered questionnaire that was divided into two sections: the first gathered demographic information, awareness of rabies, and details of bite incidents. The second part of the questionnaire explored socio-economic, logistical, and behavioural factors influencing the uptake of PEV. The questionnaire was developed through a comprehensive review of existing literature and our own expertise. Initially, it was drafted in English, then translated into Amharic, the local language, to ensure cultural relevance and clarity. Prior to full-scale data collection, a pre-test was conducted with a small sample of bite victims to assess comprehension and applicability, allowing for necessary revisions to enhance the feasibility and effectiveness. The English version of the questionnaire is attached as a supporting file (S1 File).

### Data collection method

Data was collected using a structured questionnaire to obtain quantitative information on delayed initiation of PEV, factors supposed to be affecting, and awareness of respondents to rabies. The pre-designed questionnaire was first uploaded into Kobo Toolbox, a digital data collection tool. Data collection was carried out every day for months by face-to-face interviews, in the morning from 12:00AM to 2:00PM local time, as PEV administration at Policlinic typically occurred during this period. The whole data used in this paper are attached in S1 Table.

### Data analysis

The collected data were organized using Microsoft Excel and analyzed using STATA version 17. Frequencies, percentages, and proportions were used to describe the data. Prevalence of delayed initiation of PEV was calculated as a percentage by dividing the number of rabies-exposed individuals who seek PEV after 48 hours of the exposure by the total number of individuals exposed. Analyses were performed using logistic regression models to test association between delayed initiation of PEV as the dependent variable and other possible influencing factors. Variables statistically significant at the univariable analyses were further tested in the multivariable analyses. Two variables were retained in the multivariable model; they were checked for collinearity using variance inflation factor and for interactions. However, there were no collinearity and interaction found. A P-value of <0.05 was considered significant.

## Results

This study was conducted to estimate the prevalence of delay in initiating rabies PEV and to identify potential influencing factors. The prevalence of delay in initiation of PEV was estimated to be 83.1% while only 16.9% initiated PEP within the recommended time.

Majority of respondents were males (54.62%) and aged between 18 and 30 years (48.85%). Most participants practiced Orthodox Christianity (96.54%) and had educational backgrounds above primary level (44.62%). Regarding family income, 28.85% reported monthly earnings between 3,001 (20 USD) and 6,000 (40 USD) Ethiopian Birr (Table 1).

**Table 1:** Demographic characteristics of individuals (n = 260) exposed to rabies or their caregivers in three Gondar zones of Ethiopia.

Variable	Category	Frequency	Percent (%)
**Sex**	Male	142	54.6
	Female	118	45.4
**Age in years**	18-30	127	48.8
	31-50	105	40.4
	> 50	28	10.8
**Religion**	Orthodox	251	96.5
	Other	9	3.5
**Level of education**	Cannot read and write	83	31.9
	Read or write	24	9.2
	Primary (1–8 garde)	37	14.2
	Above primary		44.6
**Monthly income of the family in Ethiopian Birr**	< 3,000	67	25.8
	3,001-6,000	75	28.8
	6,001-12,000	63	24.2
	> 12,000	55	21.2

As showed in Table 2, a significant majority of respondents (96.54%) aware rabies and recognized that it is fatal disease; 75.38% were aware of the modes of transmission, 71.54% could identify the clinical signs of rabies, and 88.85% knew that rabies exists.

**Table 2:** Awareness of rabies-exposed victims or their caregivers about rabies before exposure incidents in three Gondar zones of Ethiopia.

Variable	Category	Frequency	Percent (%)
**Know rabies exists**	Yes	231	88.8
	No	29	11.2
**Know way(s) of transmission of rabies**	Yes	196	75.4
	No	64	24.6
**Know the signs of rabies**	Yes	186	71.5
	No	74	28.5
**Know that rabies is deadly**	Yes	251	96.5
	No	9	3.5

Most of the exposures (74.62%) were by owned dogs and occurred below the waist (57.31%). owned dogs (74.62%) and occurred below the waist (57.31%). The predominant exposure type was biting (85.38%), with deep bites accounting for 40.38% of cases. Notably, 67.69% of biting animals were reported dead within 15 days post-exposure (Table 3).

**Table 3:** Characteristics of rabies exposure incidents (n = 260) in three Gondar zones of Ethiopia.

Variable	Category	Frequency	Percent (%)
Family member bitten last year	Yes	18	6.9
	No	242	93.1
**Sex of the bitten person**	Male	161	61.9
	Female	99	38.1
Age of the bitten person	<18	106	40.8
	18-30	71	27.3
	31-50	56	21.5
	> 50	27	10.4
**Ownership of biting animal**	Owned dog	194	74.6
	Stray dog	66	25.4
**Site of the bite**	Above the neck	6	2.3
	Below the neck but above the waist	78	30.0
	Below the waist	149	57.3
	Not bitten	27	10.4
Severity of exposure	Bite	222	85.4
	Contact with saliva	38	14.6
Severity of bite	Shallow bite	38	14.6
	Scratch	117	45.0
	Deep bite	105	40.4
Species of biting animal	Dog	248	95.4
	Cat	2	0.8
	Other animals	10	3.8
Status of the biting animal within 15 days after bite	Alive	26	10.0
	Dead	176	67.7
	Escaped	58	22.3

A significant majority (60%) identified the status of the biting animal as a reason for delay, while 39.6% cited distance from health facilities as a reason for delay. Despite high awareness of the need for vaccination (95.4%) and belief in its effectiveness (91.2%), 83.1% initiated PEV more than two days after exposure. Distance was not a reason for 61% of respondents for their delay in initiating PEV. Similarly, 76% of respondents reported that the cost of vaccination did not hinder them for prompt initiation of PEV and 95% agreed that the costs were fair (Table 4).

**Table 4:** Beliefs and perceptions of respondents (n = 260) about post-exposure vaccination of rabies in the three Gondar zones of Ethiopia.

Variable	Category	Frequency	Percent (%)
**Bite animal health status is a reason for delay**	Yes	156	60.0
	No	104	40.0
**Distance from health facility is reason for delay**	Yes	103	39.6
	No	157	60.4
**Knowledge of need of vaccination after exposure**	Yes	258	95.4
	No	2	0.8
**Severity of bite is reason for delay**	Yes	15	5.8
	No	245	94.2
**Costs of rabies vaccine**	500-550	208	80.0
	>550	52	20.0
**Do costs delay vaccination?**	Yes	62	23.8
	No	198	76.2
**The costs of rabies vaccine is fair**	Yes	247	95.0
	No	13	5.0
**Trained about rabies**	Yes	11	4.2
	No	249	95.8
**Belief rabies PEV is effective**	Very effective	237	91.2
	Moderately effective	23	8.9
**Effectiveness is a reason for delay**	Yes	194	74.6
	No	66	25.4
**Time to vaccinate after exposure incident**	0-1 day (48 hrs) (No delay)	36	13.9
	>1 days (48 hrs) (Delay)	224	86.1
**Opinion on the effectiveness of rabies vaccination for dogs**	Effective	243	93.5
	Not effective	17	6.5

Our logistic regression analysis has shown that from the variables tested for presence of association with delayed initiation of PEV, severity of bite and ownership of biting animal were factors associated with the outcome (Table 5).

**Table 5:** Univariable logistic regression that shows the association between possible factors and delayed initiation of rabies post-exposure vaccination in Gondar zones of Ethiopia (n = 260).

Variable	Category	Odds ratio	95% CI	P-value
Level of education	Read and write	Ref.		
	Not read or write	1.04	0.26- 4.14	0.952
	Primary (1–8)	0.45	0.11 - 1.85	0.264
	Above primary	0.61	0.17 - 2.23	0.455
Know rabies exists	Yes	Ref.		
	No	1.30	0.43 - 3.97	0.634
Know way(s) of transmission of rabies	Yes	Ref.		
	No	0.85	0.41 - 1.76	0.654
Know signs of rabies	Yes	Ref.		
	No	0.83	0.41 - 1.66	0.589
Rabies is a deadly	Yes	Ref.		
	No	1.65	0.20 - 13.57	0.639
Family member bitten in the last two years	Yes	Ref.		
	No	1.44	0.45 - 4.61	0.536
Ownership of biting animal	Owned	Ref.		
	Stray	0.41	0.20 - 0.82	0.011
Site of the bite	Above the neck	Ref.		
	Below the neck but above the waist	2.1	0.35 - 12.55	0.416
	Below the waist	2.48	0.43 - 14.3	0.309
	Not bite	6.25	0.68 - 57.90	0.107
Severity of the bite	Shallow bite	Ref.		
	Scratch	0.38	0.08 - 1.73	0.210
	Deep bite	0.16	0.04 - 0.71	0.016
Costs of rabies vaccine	500-550	5.93	4.03 - 8.74	0.000
	>551	0.46	0.22 - 0.94	0.034
Trained about rabies	Yes	Ref.		
	No	1.09	0.23 -5.25	0.909
Effectiveness of PEV is a reason for delay	Yes	Ref.		
	No	1.13	0.55 - 2.57	0.657

Note: ^a^95% confidence interval, ^b^reference category.

Multivariable logistic regression analysis had shown that deep bite (OR = 0.22, 95% CI: 0.07 - 0.66, P = 0.008) and bitten by stray dogs (OR = 0.51, 95% CI = 0.27 - 0.95, P = 0.033) reduced delay in initiating PEV (Table 6).

**Table 6:** Multivariate logistic regression that shows the association between possible factors and delayed initiation of rabies post-exposure vaccination in Gondar zones of Ethiopia (n = 260).

Variable	Category	Odds ratio	95% CI^a^	P-value
**Ownership of biting animal**	Owned	Ref.		
	Stray	0.51	0.27 - 0.95	0.033
**Severity of the bite**	Shallow bite	Ref.		
	Scratch	0.54	0.17 - 1.70	0.293
	Deep bite	0.22	0.07 - 0.66	0.008

Note ^a^95% confidence interval, ^b^Reference category.

## Discussion

Rabies is an infectious zoonotic viral disease that is almost always fatal following the onset of clinical symptoms [9]. Although rabies is 100% fatal as soon as symptoms appear, it is 100% preventable by mass vaccinating rabies-susceptible dog populations, ensuring prompt access to and effective delivery of PEV, and increasing awareness and engagement of communities who are at risk of rabies [32].

The most effective strategy available to prevent rabies in humans and livestock is preventive vaccination of dogs [29,33]. Dog vaccination coverage in Ethiopia is far lower than the standard WHO recommendation of 70% vaccination coverage in urban areas [34]. One reason for the low coverage could be the costs of vaccination. Although money is not the only factor, Beyene et al. [20] from Ethiopia, Wera et al. [35] from Indonesia, Thomas et al. [36] from South America, and Wani et al. [11] from India reported that lack of money to cover the costs of rabies vaccine is a barrier for dog owners to vaccinate their dogs against rabies. A recent work by Mengie et al. [31] from northwest Ethiopia supports this report; except few owners in urban areas, a large number of dog owners were not willing to vaccinate their dogs against rabies at their own costs. When it comes to human exposure to rabies in the current study, the great majority of respondents (95%) agree that the costs of PEV are fair and explained that costs of vaccine could not be a reason for delay to initiate the PEV.

Once exposed to a bite by a rabid animal, human rabies can only be prevented through prompt post exposure prophylaxis comprising of immediate administration of rabies vaccines, thorough washing and flushing of the wound, and if recommended, infiltration of rabies immunoglobulin (RIG) into and around the wound [10]. Thorough wound washing with detergent and water is a measure to mechanically reduce the viral inoculum at the wound site, antibodies induced by PEV lower the risk of rabies virus entering peripheral nerves from the site of bite, and if recommended, timely administration of RIG neutralizes the rabies virus at the wound site [32]. Effectiveness of antibodies induced by PEV depends on the quality of the rabies vaccine and the time at which the vaccination is initiated.

Delays in initiation of PEV often associated with higher risk of vaccine failure and rabies [37]. Our findings indicate a substantial proportion of patients initiated PEV later than recommended. In this study, delayed PEV initiation was defined as vaccination starting more than 48 hours after exposure. This threshold was chosen based on local conditions in Ethiopia, including long distances to health facilities, limited community awareness about rabies, and other access barriers. While WHO recommends need of initiation of PEV as soon as possible, setting a 48-hour cut-off might be reasonable from the local context for analyzing delays. We acknowledge that this threshold is context-specific and may affect comparability with studies from other settings and could not limit teaching the WHO standard of initiation of PEV in the health extension service. With this cut-off, we estimated 83.1% prevalence of delay in initiating PEV, which was high although it was not possible to compare the current estimate with similar reports from Ethiopia, as, to our knowledge, there were no studies so far that reported delay in initiation of PEV in the country. Our estimate of the delay in prevalence of delay in initiating PEV was higher than reports from other countries outside Africa: 27.9% reported by Khan et al. [38] from Eastern Uttar Pradesh of India, 35.5% reported by Liu et al. [39] from China, 6.8% by Esmaeilzadeh et al. [40] from southern Iran, and 10.1% reported by Singkam et al. [41] from Thailand. A possible reason for this variation could be availability and accessibility of the vaccine in these non-African countries. Delayed initiation of rabies PEV in Africa is a significant issue, with studies from countries like Uganda and Ghana. However, our estimate was also substantially higher than the prevalence of delay in initiation of PEV reported from African countries: by Kisaka et al. [42], who reported 40% from Wakiso and Kampala districts of Uganda, and by Addai and Nuertey from Ghana, who reported that only 18.7% rabies exposed individuals initiated PEV within 24 hours of bite [43]. This could be related to vaccine uptake, which was associated with multiple factors [44].

Prevention of rabies also relies heavily on the awareness of at-risk populations about the disease. In the current study, we have shown that the majority of respondents (75.4% to 96.5%) were aware existence of rabies, the ways of transmission and fatal nature of rabies, and the need of vaccination after exposure. These findings were consistent with previous reports from Ethiopia, with the work of Hagos et al. [45] from Mekele, Kebede et al [46] from Jima zone of Oromia, and Bahiru et al. [47] from Amhara region who reported, respectively, 87.8%, 95.7% and 98.4% level of awareness of individuals related to rabies. Awareness of people about risk of rabies is generally high in North West Ethiopia [31,48], but death of humans due rabies is also high [19]; we found also high prevalence of delay in initiating of PEV. Deaths may be related to delayed initiation of PEV [31]. It was reported that in Ethiopia, in general, rabies exposed victims especially those from rural areas often see traditional healers for the diagnosis and treatment for rabies exposure and seek PEV after exhausting the traditional medicinal intervention [26]. Yibrah and Damtie [49] outlined that only 23.2% of bitten victims came to the health center for seeking PEV two or more weeks after the exposure. This report is supported by Aga et al. [50] who reported that most patients who die from rabies did not receive timely and appropriate post exposure prophylaxis after bite incidents.

Related to rabies vaccine, initiation of PEV was associated with factors such as type of exposure, characteristics of the injury, health condition of the biting dog [38,40], sex and age of rabies exposed individuals or inadequate awareness about rabies severity, work-related constraints, distance of anti-rabies clinics as well as severity of wound [11,12]. In the current study, individuals who sustained deep bites were less likely to delay initiation of PEV compared with those who experienced shallow bites. This finding may be explained by the fact that severe injuries are more likely to be perceived as serious and potentially life-threatening, prompting victims to seek medical care promptly. Greater pain, tissue damage, and fear of rabies following deep bites may also increase the urgency of treatment seeking. Similarly, victims bitten by stray dogs were less likely to delay PEV initiation than those bitten by owned dogs. This could be because stray dogs are often perceived as having a higher risk of rabies infection, whereas owned dogs may be considered healthy, vaccinated, or less likely to transmit rabies. Such perceptions may lead individuals bitten by owned dogs to underestimate their risk and postpone seeking PEV. In contrast, uncertainty regarding the health status and vaccination history of stray dogs may encourage earlier healthcare-seeking behavior and timely initiation of PEV. These findings suggest that risk perception plays an important role in influencing timely uptake of rabies PEV, highlighting the need for public health messages emphasizing that all suspected rabies exposures, regardless of severity of bite or ownership of biting animal, require immediate medical evaluation.

As limitation, the study was conducted in a cross-sectional design, it was not possible to establish causal relationships. Moreover, as the study was conducted among individuals who sought rabies PEV at a health facility, it is not possible to generalize to all exposed individuals in the community, especially those who did not seek care or were unable to access vaccination services.

## Conclusion

Awareness of the community about rabies and its control is high; but, the prevalence of delayed in initiation of PEV was also high while timely and appropriate administration of PEV is crucial for neutralizing the rabies virus at the bite site and limit the rabies virus to reach the central nervous system. Therefore, it is possible to suggest that, although the level of awareness of the community, in general, was high, respondents may not have been aware of the risk of delay. These insights are vital to enhance the effectiveness of rabies control programs. Targeted interventions that promote the timely initiation of post-exposure prophylaxis vaccination are strongly recommended.

## Supporting information

S1 File Questionnaire for Delay in initiation of rabies post-exposure vaccination. (PDF)

S1 Table Data used in this paper. (XLSX)
