# Supplementary material for: Delay in initiation of rabies post-exposure vaccination and influencing factors in Gondar zones of Ethiopia
Source: PLoS Negl Trop Dis. 2026 Oct 13;20(10):e0014729. doi: 10.1371/journal.pntd.0014729 (PMC13660879; doi:10.1371/journal.pntd.0014729)
Supplement: Supplementary file 1 [file pntd.0014729.s001.pdf]

Date: ---/-----/--------

Questionnaire for Delay in initiation of rabies post-exposure vaccination

Dear respondent,

The purpose of this questionnaire is to obtain data for the research “Delay in initiation of rabies post-exposure vaccination and influencing factors in Gondar zones of Ethiopia”. The aims of the study were to estimate delay in initiating rabies PEV and to identify potential factors influencing factors for the delay in the Gondar zones of Ethiopia.

Your response is important for the success of the study. So, I kindly request your willingness to answer questions I am going to ask you. Your response will be confidential and I assure you that the data will be analyzed and reported anonymously.

Please be aware that you have the right to stop or withdraw from the study with any reason.

Thank you in advance for your willingness to participate in the study.

<u>Part I: Question on personal information</u>

1. Participant ID: ______________
2. Address: Zone ___________ District __________ Telephone no. ______________
3. Sex: ☐ Male ☐ Female
4. Age: __________
5. Religion __________________________
6. Level of education __________
7. Average monthly income of the family __________________________

<u>Part Two: Questions about the topic</u>

1. Have you ever heard of rabies before you get exposed? ☐ No ☐ Yes
2. Do you know whether rabies exists? ☐ No ☐ Yes

3. Do you know how rabies is transmitted? ☐ No ☐ Yes

24 Do you know the symptoms of rabies? ☐ No ☐ Yes

25 If the answer is yes, what are the symptoms? ___________________

26 Do you know whether rabies is fatal disease? ☐ No ☐ Yes

27 Is there any family member bitten by rabid dog/rabid animal in the last one year?

☐ No ☐ Yes

28 Was the animal owned or stray?

☐ Owned ☐ Stray

29 What was/were the sex of the bitten person? ☐ Male ☐ Female

30 What was/were the age of the bitten person? _______________

31 What was the site of bite of the bitten person? _______________

32 What was severity of exposure? ☐ Contact with saliva ☐ Bite

33 What was the severity of bite of the person?

☐ Shallow ☐ Scratch ☐ Deep bite

34 What was the status of the biting animal? ☐ Alive ☐ Dead ☐ Escaped

35 Do you think the suspected animal status be reason for delay in taking vaccination?

☐ No ☐ Yes

36 What was the species of biting animal? _______________

37 When did the person bitten by the rabid dog/animal? __________________

38 Do you believe it is necessary to be vaccinated after exposure to rabies?

☐ No ☐ Yes

39 Did/does the bitten person started vaccination? ☐ No ☐ Yes

40 How long the bitten person waited before taking vaccination after being bitten? _____________

41 How far is the nearest health service you able to get rabies vaccination from your residence:-

a. In kilometers? __________________

b. In hours of walk? ___________________,

42 Do you think severity of bite be reason for delay in taking vaccination?

☐ No ☐ Yes

43 Do you think number of bite wounds be reason for delay in taking vaccination?

☐ No ☐ Yes

44 Do you think distance of health centers a family able to get rabies vaccination from residence be reason for delay in taking vaccination? ☐ No ☐ Yes

45 What are the costs of vaccination of rabies in Ethiopian Birr? __________

46 Does your family afford the costs of vaccination for rabies?

☐ No ☐ Yes

47 Do you think the costs of vaccination be reason for delay in taking vaccination?

☐ No ☐ Yes

48 Do you think the cost of the rabies vaccine is fair compared to the benefits?

☐ No ☐ Yes

49 Do you or any of your family members get any training about rabies?

☐ No ☐ Yes

50 How effective do you think the vaccine given after exposure to rabies?

☐ Very effective ☐ moderately effective ☐ Ineffective

51 Do you think effectiveness of rabies vaccine be reason for delay in taking rabies PEP?

☐ No ☐ Yes

52 Do you lack information where you get rabies vaccination? ☐ No ☐ Yes

53 Do you believe that vaccinating dogs against rabies is an effective way to prevent the spread of the disease? ☐ No ☐ Yes

54 How confident are you about the effectiveness of rabies vaccine given after exposure to rabies?

☐ Very confident ☐ moderately confident ☐ Don't believe it is effective

Thank you for your willingness in answering the questions.

Good bye
